# Delineation of proteome changes driven by cell size and growth rate

**DOI:** 10.3389/fcell.2022.980721

**Published:** 2022-09-05

**Authors:** Evgeny Zatulovskiy, Michael C. Lanz, Shuyuan Zhang, Frank McCarthy, Joshua E. Elias, Jan M. Skotheim

**Affiliations:** ^1^ Department of Biology, Stanford University, Stanford, CA, United States; ^2^ Chan Zuckerberg Biohub, Stanford, CA, United States

**Keywords:** cell size, growth rate, quantitative proteomics, mTOR, rapamycin, protein synthesis rate, senescence

## Abstract

Increasing cell size drives changes to the proteome, which affects cell physiology. As cell size increases, some proteins become more concentrated while others are diluted. As a result, the state of the cell changes continuously with increasing size. In addition to these proteomic changes, large cells have a lower growth rate (protein synthesis rate per unit volume). That both the cell’s proteome and growth rate change with cell size suggests they may be interdependent. To test this, we used quantitative mass spectrometry to measure how the proteome changes in response to the mTOR inhibitor rapamycin, which decreases the cellular growth rate and has only a minimal effect on cell size. We found that large cell size and mTOR inhibition, both of which lower the growth rate of a cell, remodel the proteome in similar ways. This suggests that many of the effects of cell size are mediated by the size-dependent slowdown of the cellular growth rate. For example, the previously reported size-dependent expression of some senescence markers could reflect a cell’s declining growth rate rather than its size *per se*. In contrast, histones and other chromatin components are diluted in large cells independently of the growth rate, likely so that they remain in proportion with the genome. Finally, size-dependent changes to the cell’s growth rate and proteome composition are still apparent in cells continually exposed to a saturating dose of rapamycin, which indicates that cell size can affect the proteome independently of mTORC1 signaling. Taken together, our results clarify the dependencies between cell size, growth, mTOR activity, and the proteome remodeling that ultimately controls many aspects of cell physiology.

## Introduction

The importance of cell size is reflected in its uniformity within any given cell type. Deviations from a typical size are often associated with disease states, such cancer and aging (Ginzberg et al., 2015; Li et al., 2015; Nguyen et al., 2016; Neurohr et al., 2019; Zatulovskiy and Skotheim, 2020; Cheng et al., 2021; Lengefeld et al., 2021; Lanz et al., 2022; Sandlin, 2022). To avoid such problematic disease states, eukaryotic cells rely on dedicated mechanisms that correct for deviations from their target cell size (Ginzberg et al., 2015; Cadart et al., 2018; Ginzberg et al., 2018; Liu et al., 2018; Zatulovskiy et al., 2020; Zatulovskiy and Skotheim, 2020).

**FIGURE 1:**
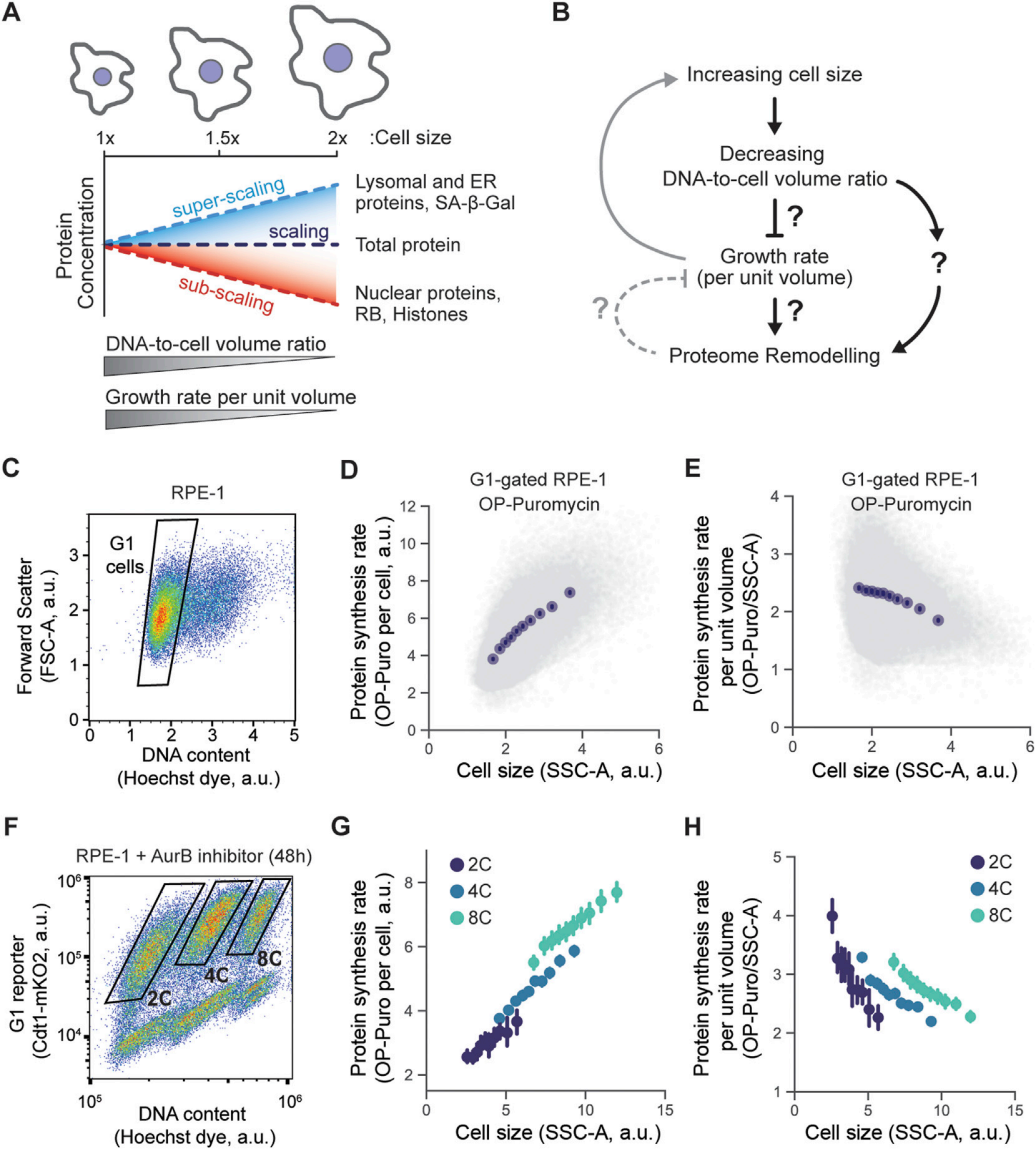
The relationship between cell size and growth rate. **(A,B)** Schematics depicting the phenotypes associated with increasing cell size. Increase in cell size leads to the decrease in DNA to cell volume ratio and reduction of cell growth rate per unit volume. Changes in cell size are also accompanied by global proteome remodeling, i.e., some proteins are concentrated while others are diluted as cells grow larger. **(C-E)** Protein synthesis rate measured using OP-puromycin incorporation. After 30 min labeling, OP-puromycin was click-conjugated to a fluorescent azide and measured using flow cytometry. RPE-1 cells were gated for G1 DNA content **(C)**, and the incorporated OP-puromycin amount, which reflects the nascent protein synthesis rate, was plotted against the Side Scatter (SSC-A), a proxy for cell size **(D)**. To represent the synthesis rate per unit volume, the conjugated OP-puromycin fluorescence intensity was divided by the cell volume proxy SSC-A and plotted against the SSC-A **(E)**. **(F-H)** Same experiment performed in **(C–E)** but measuring the protein synthesis rates in populations of RPE-1 cells with different ploidies. To generate tetraploid and octoploid cells, the cytokinesis was partially inhibited in RPE-1 cells using the Aurora kinase B inhibitor barasertib (100 nM, 48 h) (Mu et al., 2020; Lanz et al., 2022), then 2C, 4C, and 8C were then gated using flow cytometry **(F)**. The overall protein synthesis rate **(G)** and the protein synthesis rate per unit volume **(H)** were plotted against the SSC-A, a proxy for cell volume. Each experiment was performed in *n* = 3 biological replicates, and a representative example for each experiment is shown in this Figure. Data in plots **(D)**, **(E)**, **(G)**, and **(H)** were binned by cell size (SSC-A), and means ±95% confidence intervals were plotted for each bin.

The relationship between cell size and cell physiology is apparent from size-dependent changes to the proteome. While most protein concentrations remain fairly constant as cells grow larger (protein amounts scale in proportion to cell size), many others increase (superscaling proteins) or decrease (subscaling proteins) in concentration (Chen et al., 2020; Cheng et al., 2021; Claude et al., 2021; Lanz et al., 2022) (Figure 1A). Although a small change in a single protein’s concentration may not substantially affect cell physiology, the cumulative effect of thousands of such changes could account for profound changes in cell physiology. For example, recent work shows that excessively large cell size promotes senescence (Demidenko and Blagosklonny, 2008; Neurohr et al., 2019; Lengefeld et al., 2021; Wilson et al., 2021; Crozier et al., 2022; Lanz et al., 2022). Thus, while differential size scaling of protein concentrations likely has important implications for cell physiology, the mechanistic origins of such size scaling remain unknown. In other words, how do increases in cell size result in size-dependent changes of protein concentrations?

One possibility for how cell size drives changes in protein concentrations would be that this is an indirect effect through the cell’s protein synthesis rate per unit volume (hereby referred to as growth rate), which decreases in larger cells (Cadart et al., 2018; Neurohr et al., 2019; Liu et al., 2022) (Figure 1B). There seems to be a limit to the size range of effective biosynthesis, with larger cells exhibiting a loss of mitochondrial membrane potential (Miettinen and Björklund, 2016) and reduced proliferative capacity (Demidenko and Blagosklonny, 2008; Cheng et al., 2021; Lengefeld et al., 2021; Wilson et al., 2021; Lanz et al., 2022). As cells get larger, their growth becomes less efficient, and this may affect the concentrations of diverse sets of proteins. Another possibility is that cell size directly affects protein concentrations independent of the cell’s growth rate. Because the size-scaling behavior of each individual protein is in part predicted by its synthesis efficiency and degradation rate (Lanz et al., 2022), we hypothesized that the effects of cell size on the proteome may be mediated by the decrease in growth rate observed in larger cells.

Here, we investigate how cell size and growth rate account for measurable differences in protein concentrations. We found that a decrease in global protein synthesis rates induced with the mTOR inhibitor rapamycin remodels the proteome to resemble that found in large cells. However, among rapamycin-treated cells we still observe size-dependent changes to the proteome similar to those found in untreated cells. This indicates that many size-dependent changes to protein concentrations are mediated by growth slowdown in large cells, which can occur independently of mTORC1 signaling. Moreover, we identify specific proteome changes that occur only in large cells or only upon rapamycin treatment. Thus, our work identifies both growth-dependent and size-dependent contributions to the proteome's size-dependence.

## Materials and methods

### Cell culture

Telomerase-immortalized retinal pigment epithelium cells (*hTERT RPE-1*, here also referred to as RPE-1 for brevity) were obtained from the Cyert laboratory at Stanford. All cells were cultured at 37°C with 5% CO_2_ in Dulbecco’s modification of Eagle’s medium (DMEM) with l-glutamine, 4.5 g/L glucose and sodium pyruvate (Corning), supplemented with 10% heat-inactivated fetal bovine serum (FBS, Corning) and 1% penicillin/streptomycin.

### Fluorescence-activated cell sorting

Fluorescence-activated cell sorting (FACS) was used to sort rapamycin-treated RPE-1 cells by their size and cell cycle phase. To do this, the cells were cultured for 48 h in the presence of 20 nM rapamycin, then harvested from dishes by trypsinization, stained with 20 µM Hoechst 33342 DNA dye in PBS for 15 min at 37°C, and then sorted on a BD FACSAria Fusion flow cytometer. Consecutive SSC-A over FSC-A, and FSC-H over FSC-A gates were used to isolate single cells. Then, G1 cells were gated by DNA content (Hoechst 33342 staining). Finally, we collected the 10% smallest and 10% largest cells, as well as another 10% of the cells near the average size, using the gating based on SSC-A signal, as in (Lanz et al., 2022). During sorting, all cell samples and collection tubes were kept at 4°C. To determine the cell size distributions of the collected samples, aliquots were taken from each sorted size bin and measured on a Z2 Coulter counter (Beckman). Sorted cells were then spun down, lysed in RIPA buffer on ice and used for subsequent proteomics analysis.

### Protein synthesis rate measurement

To calculate the rate of protein synthesis, RPE-1 cells were pulse-labeled with a puromycin analog O-Propargyl-puromycin (OP-puromycin), which incorporates into nascent polypeptide chains and terminates their translation (OP-puromycin was purchased from ClickChemistryTools, Cat 1407). RPE-1 cells were plated 1 day before labeling and reached ∼70% confluence by the time of labeling. Cells were labeled with 20 μM OP-puromycin (final concentration) for 30 min. Then cells were trypsinized, fixed with 3% paraformaldehyde, and permeabilized with 90% ice-cold methanol. Click reaction was performed using the Click-&-Go Cell Reaction Buffer kit (ClickChemistryTools, 1263) with AZDye-488 azide (ClickChemistryTools, 1275). Following the Click-reaction and PBS washes, cells were then stained with DAPI and analyzed using Flow Cytometry. In polyploidy experiments, RPE-1 cells expressing a FUCCI cell cycle reporter (Sakaue-Sawano et al., 2008) were used, which allowed us to gate out 2C, 4C, and 8C G1-phase cells based on the Cdt1-mKO2 marker fluorescence and the DNA content.

### LC-MS/MS sample preparation

RPE-1 cells were lysed in RIPA buffer on ice, and cell lysates were cleared by centrifugation at 15000xg for 30 min at 4°C. Lysates were then denatured in 1% SDS and reduced with 5 mM DTT (10 min at 65°C), then alkylated with 7.5 mM iodoacetamide (15 min at room temperature), and precipitated with three volumes of a solution containing 50% acetone and 50% ethanol (with 0.1% Acetic acid). Proteins were re-solubilized in the buffer containing 2 M urea, 50 mM Tris-HCl, and 150 mM NaCl. Re-solubilized proteins were then digested with TPCK-treated trypsin (50:1) overnight at 37°C. Peptides were desalted using 50 mg Sep-Pak columns. 20 µg of dried peptides were resuspended in 20 µl of 100 mM TEAB for TMT labeling reaction. Our method for TMT labeling was adapted from Zecha et al. (2018) and the Thermo TMT10plex™ Isobaric Label Reagent Set Protocol. 20µg of peptide was labeled using 100 µg of Thermo TMT10plex™ in a reaction volume of 25 µl for 1 h. The labeling reaction was quenched with 8 µL of 0.5% hydroxylamine for 15 min. Labeled peptides were pooled, acidified to a pH of ∼2 using drops of 10% TFA, and desalted again with a Sep-Pak 50 mg C18 column as described previously (Lanz et al., 2022).

### High-pH reverse phase fractionation

TMT-labeled peptides were fractionated using a Pierce™ High pH Reversed-Phase Peptide Fractionation Kit. Peptides collected from eight fractions were dried and reconstituted in 0.1% formic acid.

### LC-MS/MS data acquisition

Fractionated TMT-labeled peptides were analyzed on a Fusion Lumos mass spectrometer (Thermo Fisher Scientific, San Jose, CA) equipped with a Thermo EASY-nLC 1200 LC system (Thermo Fisher Scientific, San Jose, CA). Peptides were separated by capillary reverse phase chromatography on a 25 cm column (75 µm inner diameter, packed with 1.6 µm C18 resin, AUR2-25075C18A, Ionopticks, Victoria Australia). Electrospray Ionization voltage was set to 1550 V. Peptides were resuspended in 10 µl of 0.1% formic acid. 2 µl was introduced into the Fusion Lumos mass spectrometer using a two-step linear gradient with 6–33% buffer B (0.1% (v/v) formic acid in 80% acetonitrile) for 145 min followed by 33–45% buffer B for 15 min at a flow rate of 300 nL/min. Column temperature was maintained at 40°C throughout the procedure. Xcalibur software (Thermo Fisher Scientific) was used for the data acquisition and the instrument was operated in data-dependent mode. Survey scans were acquired in the Orbitrap mass analyzer over the range of 380–1800 m/z with a mass resolution of 70,000 (at m/z 200). Ions were selected for fragmentation from the 10 most abundant ions with a charge state of either 2, 3 or 4 and within an isolation window of 2.0 m/z. Selected ions were fragmented by Higher-energy Collision-induced dissociation (CID) with normalized collision energies of 35%, and the tandem mass spectra were acquired in the Ion trap mass analyzer with a “Rapid” scan rate. Repeated sequencing of peptides was kept to a minimum by dynamic exclusion of the sequenced peptides for 60 seconds. For MS/MS, the AGC target was set to “Standard” and max injection time was set to 35 ms. Relative changes in peptide concentration were determined at the MS3-level by isolating and fragmenting the 5 most dominant MS2 ion peaks using HCD. TMT reporter ions were resolved in the orbitrap at a resolution of 50,000.

### Spectral searches

All raw files were searched using the Andromeda engine (Cox et al., 2011) embedded in MaxQuant (v1.6.7.0) (Cox and Mann, 2008). Reporter ion MS3 search was conducted using 10plex TMT isobaric labels. Variable modifications included oxidation (M) and protein N-terminal acetylation. Carbamidomethyl (C) was a fixed modification. The number of modifications per peptide was capped at five. Digestion was set to tryptic (proline-blocked). Database search was conducted using the UniProt proteome - Human_UP000005640_9606. Minimum peptide length was seven amino acids. FDR was determined using a reverse decoy proteome (Elias and Gygi, 2007).

### Peptide quantitation

Our TMT analysis pipeline uses the peptide feature information in MaxQuant’s “evidence.txt” output file. Each row of the “evidence.txt” file represents an independent MS3-level TMT measurement. Contaminant and decoy peptide identifications were discarded. Peptides without signal in any of the TMT channels were also excluded. TMT peptide measurements were assigned to proteins based on MaxQuant’s “Leading razor protein” designation. For each peptide, the fraction of ion intensity in each TMT channel was calculated by dividing the “Reporter ion intensity” column by the sum of all reporter ion intensities. TMT channels were normalized by adjusting the fraction of ion intensity in each channel by the median for all measured peptides. Changes in the concentration of a protein were determined by the median concentration change for all its peptides. Protein Slope values were calculated as described previously (Lanz et al., 2022).

### 2D annotation enrichment analysis

Annotation enrichment analysis in Figure 6 was performed as described previously (Cox and Mann, 2012). The protein annotation groups were deemed significantly enriched and plotted in Figure 6 if the Benjamini-Hochberg FDR was smaller than 0.02. The position of each annotation group on the plot is determined by the enrichment score (S). The enrichment score is calculated from the rank ordered distribution of Protein Slope and Rapamycin ratio values:

$$\text{S}=2\left({\text{R}}_{\text{group}}-{\text{R}}_{\text{remaing proteins}}\right)/\text{n}$$

Where R_group_ and R_remaing proteins_ are the average ranks for the proteins within an annotation group and all remaining proteins in the experiment, respectively, and n is the total number of proteins. Groups of annotations colored in Figure 6 were manually curated (see Table S2).

### Principle component analysis

PCA analysis was performed in Python using the sklearn package. A data frame was created that contained individual proteins as rows with columns corresponding to the relative protein concentration in each TMT channel (obtained from the median of all peptide measurements for a given protein). For principal component analyses in Supplementary Table S3, S4, the cell size and rapamycin treatment datasets were both filtered for proteins with at least eight independent peptide measurements to increase quantitative accuracy for individual proteins.

### Protein annotations

Protein annotations in Figures 3D,E were based on UniProt columns named “Subcellular location [CC]” or “Protein names”. Protein localization was strictly parsed so that each annotated protein belongs to only one of the designated groups. Proteins with 2 or more of the depicted annotations were ignored (except for the “Cytoplasm/Nucleus” category, which required a nuclear and cytoplasmic annotation).

## Results

### Protein synthesis rate per unit volule decreases with decreasing DNA-to-cell volume ratio

Increasing cell size results in large-scale remodeling of the proteome (Lanz et al., 2022). Larger cells also have a lower protein synthesis rate per unit volume (hereby referred to as growth rate) (Tzur et al., 2009; Cadart et al., 2018; Neurohr et al., 2019; Liu et al., 2022), so that it is unclear if the size-dependent remodeling of the proteome that we have recently reported (Lanz et al., 2022) was due to changing growth rates or due to cell size *per se*. We therefore sought to determine if and how the cell’s growth rate contributes to proteome remodeling (Figure 1B).

To assess the effect of a cell’s growth rate on its proteome, we first sought to quantitatively examine the size-dependence of the protein synthesis rate in *hTERT*-immortalized RPE-1 cells. We chose to examine RPE-1 cells because they are commonly used in studies of cell growth and division. To measure protein synthesis rates, we pulse-labeled cells with a puromycin analog O-Propargyl-puromycin (OP-puromycin), which incorporates into nascent polypeptide chains and can be subsequently click-conjugated to fluorescent azides. The incorporation of OP-puromycin into a cell, which is a readout for the cell’s overall protein synthesis rate, was then measured by flow cytometry. We used the Side Scatter (SSC-A) parameter as a metric of cell size as it has been previously proven to be a good proxy for the cell volume (Tzur et al., 2011; Lanz et al., 2022). We found that the absolute protein synthesis rates were higher in larger G1 cells, consistent with the general increase in cellular biosynthesis in larger cells (Figures 1C,D). However, this increase in protein synthesis rate is outpaced by the increase in cell size so that the growth rate (protein synthesis per unit volume) is lower in larger cells, as observed previously (Cadart et al., 2018; Neurohr et al., 2019; Liu et al., 2022) (Figure 1E).

While we observe a decrease in cell growth rate with increasing cell size among diploid G1 cells, it has been shown recently that even very large cells can maintain efficient growth if the increase in cell size coincides with a proportional increase in ploidy (Mu et al., 2020). Moreover, our own work has demonstrated that size-dependent changes to the proteome are attenuated if cell ploidy is also increased in proportion to the cell volume (Lanz et al., 2022). We therefore hypothesized that the size-dependent decrease in protein synthesis rate per unit volume is determined by the DNA-to-cell volume ratio, rather than by cell size itself. To test this, we induced polyploidization in RPE-1 cells by treating them with Aurora kinase B inhibitor barasertib (as in Mu et al., 2020). We then measured OP-puromycin incorporation rates in diploid (2C), tetraploid (4C), and octoploid (8C) cells (Figures 1F–H). Like before, the protein synthesis rate per unit volume decreased with cell size within each of the 2C, 4C, and 8C populations of cells. However, when we compared similar-sized cells with different ploidies we found that increases in ploidy counteracted the decline in protein synthesis per unit volume in large cells. We therefore conclude that growth rate in cells decreases in proportion to the DNA-to-cell volume ratio.

### Partial cell growth inhibition with rapamycin leads to global proteome remodeling

**FIGURE 2:**
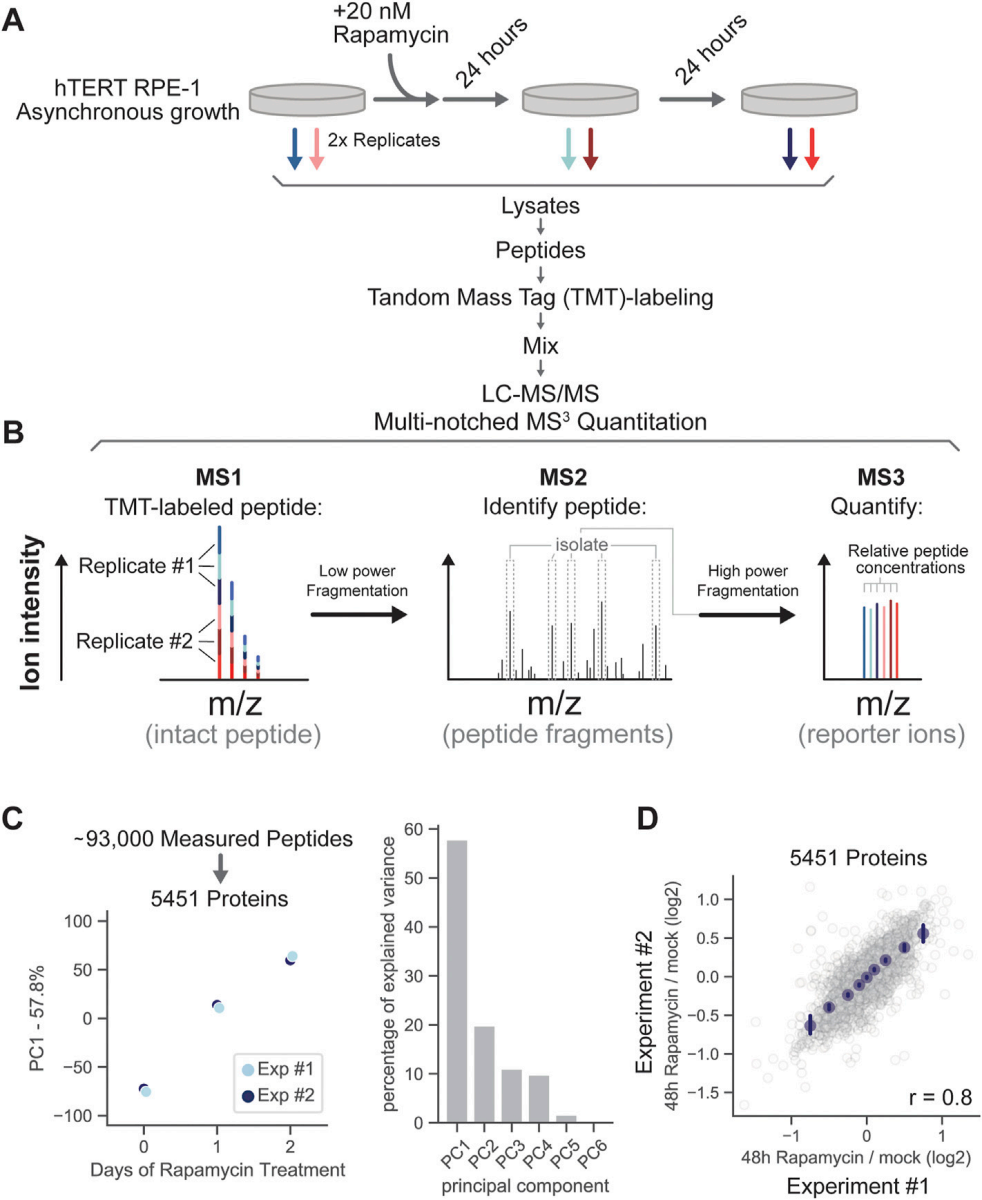
Proteome remodeling in response to mTOR inhibition. **(A)** Asynchronously growing RPE-1 cells were treated with 20 nM rapamycin for 24 and 48 h and subjected to multiplexed proteomic analysis. **(B)** Diagram outlining TMT-labeled peptide quantitation *via* multi-notched MS3 mass spectrometry. Peptides labeled with tandem mass tags (TMT) are fragmented with low collisional energy between MS-level 1 and 2 to break peptide bonds. The fragmentation steps are depicted by the two horizontal arrows. Prominent fragment ions in the MS2 spectra are then collected and fragmented with high energy to release the TMT reporter ions (MS3), which are then used to quantify changes in peptide concentration. **(C)** Principal component analysis of the relative changes in protein concentration after 0, 24, and 48 h of rapamycin treatment. **(D)** Correlation of relative protein concentration changes in replicate experiments (48 h of rapamycin treatment/0 h of rapamycin treatment). Binned average values are shown in navy blue dots and the error bars represent 99% confidence intervals. Pearson’s r value is displayed on the bottom right of the figure panel. Measurements for each individual protein can be found in Supplementary Table S1.

To disentangle the effects of cell size and cell growth rate on the proteome, we treated RPE-1 cells with a saturating dose of the mTOR inhibitor rapamycin for 24 or 48 h (20 nM, see Supplementary Figure S1) and used Tandem Mass Tag (TMT) proteomics to directly compare the relative concentrations of thousands of proteins across these time points (Figure 2A; Supplementary Table S1). Although mTOR inhibition reduces cell growth rate (Fingar et al., 2002; Wang and Proud, 2006; Tee, 2018), it had only a minimal effect on cell size in our experiments (Supplementary Figure S2) because the cells compensated for the decreased growth rate by reducing the cell division rate (Liu et al., 2018; Tan et al., 2021). We collected and lysed rapamycin-treated and untreated cells for simultaneous proteomic analysis. All proteins from the lysates were then digested into peptides using trypsin. Peptides originating from each of the six cell cultures (three experimental conditions, two biological replicate experiments) were labeled with TMT reagents, mixed together, and quantified together using a tribrid mass spectrometer (Figure 2B). The relative changes in protein concentrations were then inferred from the measurement of their peptides (Lanz et al., 2022).

We measured the relative concentrations of 5,451 proteins in response to rapamycin treatment (Figures 2C,D). Changes to the proteome between untreated cells and 24 h treatment were similar in magnitude to those between 24 and 48 h (Figure 2C), and very few proteins either increased or decreased in concentration by more than 2-fold throughout the time course (Figure 2D). While rapamycin treatment increased the fraction of G1 cells, this increase was slight and therefore unlikely to be responsible for the proteomic changes throughout the time course (Supplementary Figure S3). Replicate experiments were highly correlated (Pearson’s r = 0.8) (Figure 2D; Supplementary Table S1).

### mTOR inhibition causes proteomic changes similar to those observed in larger cells

**FIGURE 3:**
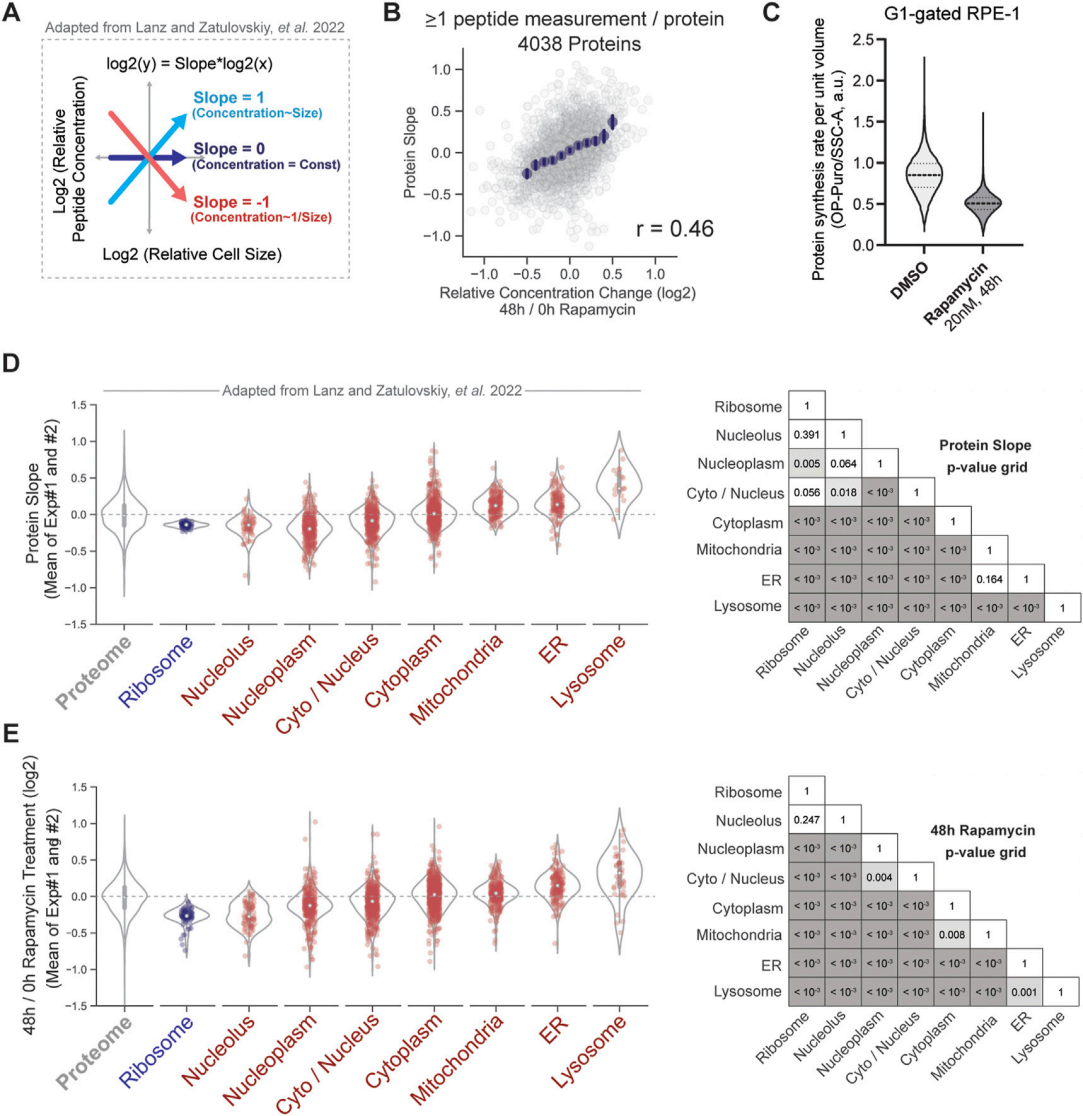
Similarities in how the proteome changes with cell size and with mTOR inhibition. **(A)** Protein slope value describes how the concentrations of individual proteins scale with the volume of the cell, as in Lanz et al., 2022. Proteins with a slope of 0 maintain a constant cellular concentration regardless of cell volume. A slope value of 1 corresponds to an increase in protein concentration that is proportional to the increase in volume, and a slope of −1 corresponds to dilution (1/volume). **(B)** Cell size-dependent changes to the proteome strongly correlate with proteome changes induced by 48 h of rapamycin treatment. Binned average values are shown in navy blue dots, and the error bars represent 99% confidence intervals. **(C)** Protein synthesis rate per unit volume measured in rapamycin-treated and untreated G1 RPE-1 cells. OP-puromycin incorporation was used as readout for protein synthesis rate, and Side Scatter as a proxy for cell volume, as in Figure 1. Violin plots show the distribution of OP-puromycin/SSC-A ratios, with dashed lines showing the medians and 25th and 75th percentiles. **(D,E)** Increase in cell size **(D)** and mTOR inhibition **(E)** elicit similar changes in organelle protein content. Violin plots depict the distribution of slope **(D)** or concentration ratio **(E)** values for each group of proteins. Individual proteins in each annotation group are plotted as dots. The *p*-values comparing the slopes **(D)** or ratios **(E)** for every pair of protein groups within the same experiment are visualized in a grid format. Proteins are non-redundantly parsed by subcellular localization using UniProt annotations (see Methods).

To investigate how the cellular growth rate contributes to the proteome changes previously associated with cell size, we compared our new proteome dataset for rapamycin treated cells with our recently published proteomic dataset for G1 cells of different sizes (Lanz et al., 2022). To describe how the concentrations of individual proteins change with cell size, we use the “Protein Slope” value (as described in Lanz et al., 2022) (Figure 3A). In brief, the Protein Slope is calculated from a linear regression between the log_2_ of an individual protein’s concentration and the log_2_ of the cell volume. A Protein Slope value of 0 describes proteins for which concentration does not change with cell volume (scaling); a Protein Slope value of 1 describes proteins for which concentrations increase proportionally to cell volume (superscaling); and protein slope of −1 describes proteins that are perfectly diluted by cell growth so that their concentration is inversely proportional to cell volume (subscaling).

The changes to the proteome in response to increasing cell size were very similar to those measured for rapamycin treatment. The slopes describing concentration size-dependence and the concentration fold-changes induced by mTOR inhibition were highly correlated (Pearson’s r = 0.46, *p* < 10^−3^) (Figure 3B), which is consistent with both large cell size and rapamycin treatment similarly reducing the cell growth rate by about 1.7-fold (Figure 3C). We note that both experiments examining the proteomes of rapamycin-treated and cell-size-sorted cells were performed using RPE-1 cells. The correlation between the datasets increases with a requirement for more peptide measurements per protein (Supplementary Figure S4A). These similarities between size-dependent and rapamycin-induced proteome changes are particularly apparent when proteins are grouped by subcellular localization as was done in Lanz et al. (2022) (Figures 3D,E). Moreover, we have previously shown that cell size-dependent changes to the proteome observed in RPE-1 cells are similar to those taking place in primary human lung fibroblasts (HLFs), which suggests that most of the scaling relationships we are describing are not cell type-dependent (Lanz et al., 2022).

### Size-dependent changes to the proteome persist in rapamycin-treated cells

**FIGURE 4:**
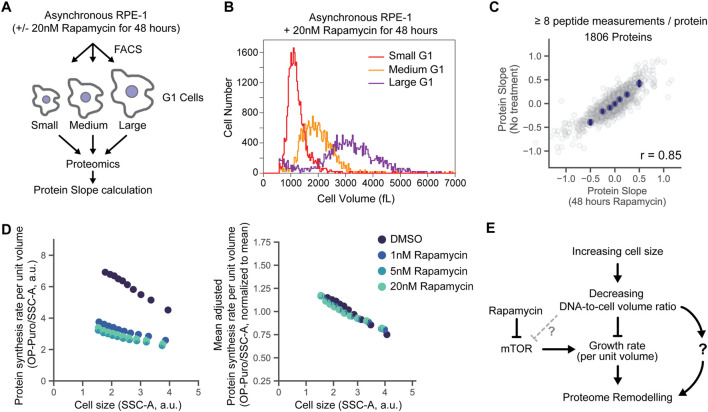
Size-dependent changes to the proteome persist in mTOR inhibited cells. **(A)** Measurement of size-dependent changes to the proteome in cells pre-incubated with rapamycin. Protein Slope calculations were determined as described previously, see Figure 3A (Lanz et al., 2022). **(B)** A representative example of cell size distributions for rapamycin-treated (20nM, 48 h) G1-phase RPE-1 cells sorted into three size bins by FACS and measured on a Coulter counter. **(C)** Correspondence of proteome scaling in the presence and absence of a saturating dose of the mTOR inhibitor rapamycin. Binned average values are shown in navy blue dots and the error bars represent 99% confidence intervals. Pearson’s correlation coefficient r is shown in the bottom right. **(D)** Size-dependent changes in protein synthesis rate per unit volume in untreated and rapamycin-treated RPE-1 cells measured by OP-puromycin incorporation, as in Figure 1E. Right hand panel shows protein synthesis rates per unit volume normalized to the mean values for each condition for easier side by side comparison. **(E)** Updated schematic depicting the possible relationships between cell size, cell growth, and mTOR and their effect on the proteome.

That mTOR inhibition led to protein concentration changes similar to those that took place in larger cells suggested that declining mTOR activity in large cells may be responsible for the size-dependent changes to the proteome that we previously reported (Lanz et al., 2022). To test this, we treated cells with a saturating dose (20 nM) of rapamycin for 48 h, then sorted G1 cells by size into small, medium, and large cell size populations using fluorescence-activated cell sorting (FACS) (Figures 4A,B). We then measured protein concentration changes across the proteome as a function of G1 cell size and calculated Protein Slopes for each protein as described above (Lanz et al., 2022) (Figure 3A). The slopes describing size-dependence of protein concentrations were similar in both untreated and rapamycin-treated cells (Pearson’s r = 0.85, *p* < 10^−3^) (Figure 4C). This experiment suggests that while mTOR is generally an important contributor to proteome remodeling, much of the proteome’s size-dependence is independent of mTOR.

To test whether the size-dependent decrease in protein synthesis rate per unit volume is dependent on the mTOR signaling, we performed an OP-puromycin incorporation assay in rapamycin-treated RPE-1 cells. We found that larger rapamycin-treated cells still had a lower protein synthesis rate per unit volume compared to their smaller counterparts (Figure 4D). This indicates that cell size reduces the growth rate per unit volume independently of mTOR signaling (Figure 4E). Alternatively, this could be partially explained by the fact that rapamycin efficiently inhibits only the mTOR complex 1 (mTORC1), while the mTOR complex 2 retains some its activities (Saxton and Sabatini, 2017; Yang et al., 2021).

### Senescence markers differentially respond to mTOR inhibition and increasing cell size

While cellular senescence has long been associated with large cell size, it was thought that this was a consequence of continued growth during the irreversible cell cycle arrest that defines the senescent state (Sharpless and Sherr, 2015; Hernandez-Segura et al., 2018). However, recent work has questioned the direction of causality and established that large cell size is not just a consequence of senescence but also contributes to cell cycle arrest (Demidenko and Blagosklonny, 2008; Neurohr et al., 2019; Lengefeld et al., 2021; Lanz et al., 2022). This relationship between cell size and senescence can also be seen in the proteome. In fact, hallmarks of senescence are increasingly pronounced in larger proliferating cells (Lanz et al., 2022), suggesting that cells continually approach a senescent state as they grow larger. Consistent with this view that large size promotes senescence, the inhibition of growth by rapamycin protects cells from becoming excessively large and senescent (Demidenko et al., 2009; Lengefeld et al., 2021; Lanz et al., 2022).

**FIGURE 5:**
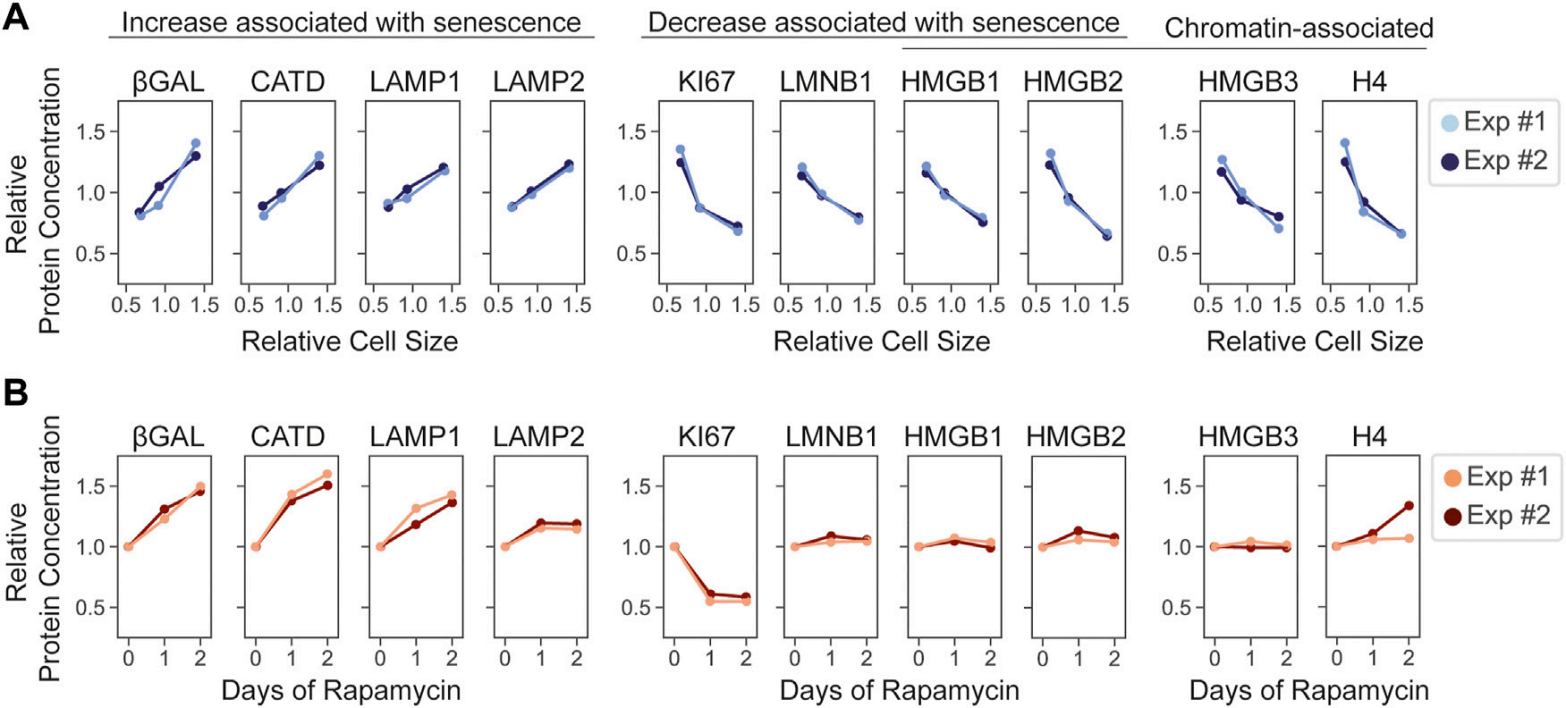
The effects of cell size and mTOR inhibition on reporters of cellular senescence. Cell-size-dependent **(A)** and rapamycin-induced **(B)** changes in the relative concentration of the indicated proteins. Two replicate experiments are depicted. For **(B)**, protein concentration is plotted relative to the Day 0 of the rapamycin time course described in Figure 2.

While large cell size is increasingly accepted as promoting senescence, the effect of rapamycin on preventing senescence is questioned by our proteomic analysis. We found that rapamycin treatment remodeled the proteome, making it *more similar* to larger cells approaching the senescent state. Yet, rapamycin treatment clearly promotes the ability of cells to reenter the cell cycle following a long cell cycle arrest (Demidenko and Blagosklonny, 2008; Demidenko et al., 2009; Leontieva and Blagosklonny, 2014; Wilson et al., 2021; Lanz et al., 2022). This paradox raises the question as to how the specific markers of senescence are changing following rapamycin exposure. To address this question, we examined how a set of commonly used markers of senescence responded to increasing cell size and to rapamycin treatment (Hernandez-Segura et al., 2018) (Figure 5). The results were mixed. Markers of senescence whose concentrations increase with cell size (superscaling), including lysosomal proteins, also increased in concentration during rapamycin exposure. The subscaling proliferative marker, Ki67, decreased in response to rapamycin treatment too (Figure 5). However, some subscaling markers, such as HMGB1 and HMGB2, whose concentrations decrease in senescent cells and in large proliferating cells, remained at constant concentration during rapamycin treatment. Thus, some markers of senescence respond to rapamycin while others do not.

Some senescence markers whose changes were specific to increasing cell size, including HMBG1 and HMGB2, were chromatin associated. That these concentrations were diluted in larger cells but not by rapamycin treatment suggests that the expression of these proteins is tied to the DNA-to-cell volume ratio, as has been recently described for histone proteins (Claude et al., 2021; Swaffer et al., 2021). We therefore examined if other chromatin components behaved similarly. Indeed, the concentrations of chromatin proteins, such as histone H4 and HMGB3, which is not linked to senescence, did not change upon rapamycin exposure (Figure 5).

It remains unclear how individual markers of senescence impact the irreversible exit from the cell cycle, which is the determining feature of senescence in proliferating cells (Sharpless and Sherr, 2015). Our analysis suggests that these markers may reflect different aspects of cell physiology such as the growth rate (e.g., β-galactosidase) or the DNA-to-cell volume ratio (e.g., HMGB proteins), both of which correlate with the onset of senescence.

### Global analysis of how the proteome changes with cell size and mTOR inhibition

**FIGURE 6:**
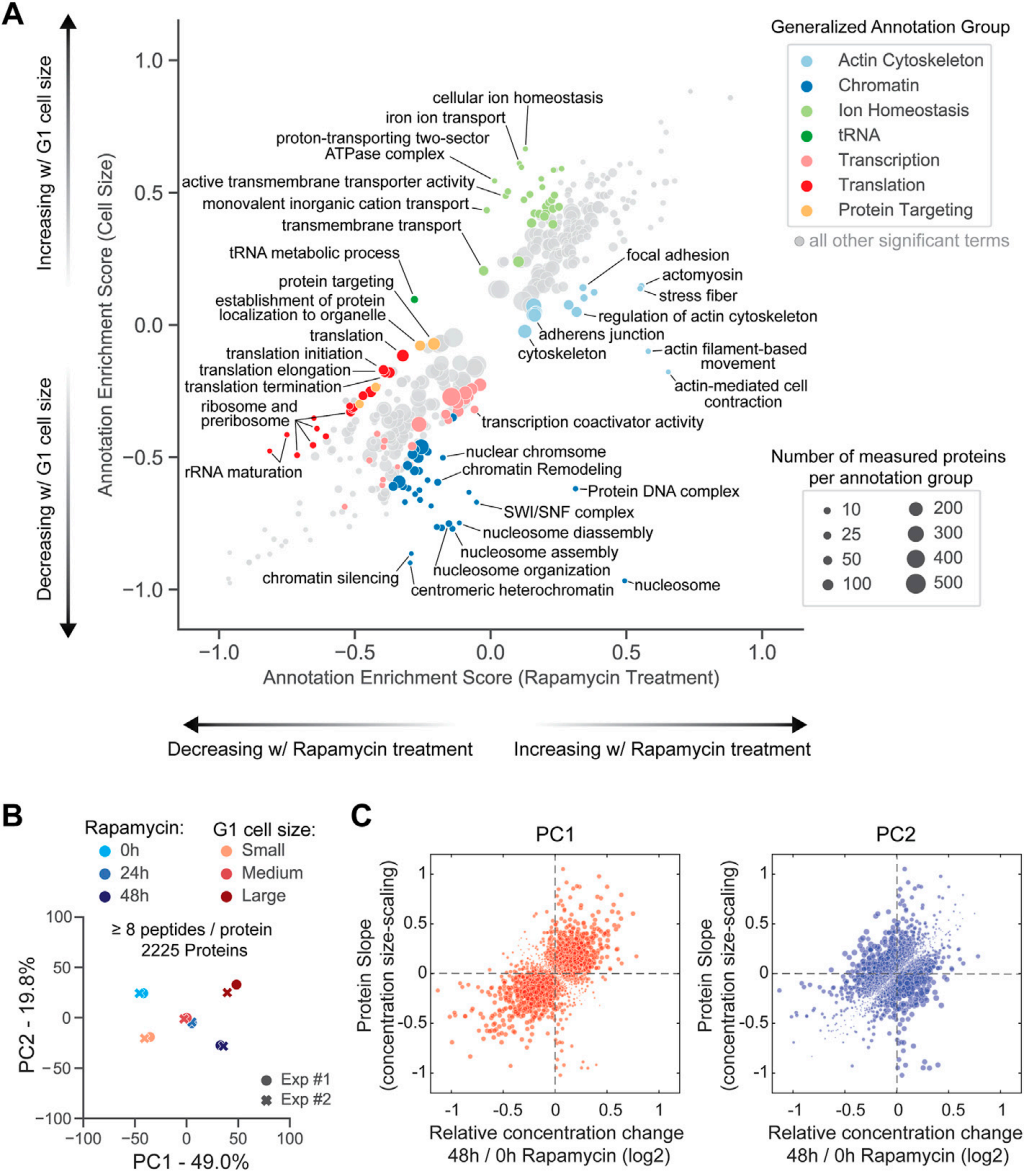
Differences in how the proteome changes with cell size and with mTOR inhibition. **(A)** 2D annotation enrichment analysis of cell-size- and rapamycin-induced proteome changes (see Methods and Supplementary Table S2). The protein annotation groups were deemed significantly enriched and displayed on the plot if the Benjamini-Hochberg FDR was smaller than 0.02. Each dot represents a group of annotated proteins. The size of the dot corresponds to the number of proteins within that annotation group, and the dot’s position along the axes represents the degree to which those proteins' concentrations are changing in both datasets (based on rank ordering). Protein groups occupying the 4 sectors that deviate most from the x = y diagonal were manually curated into a larger “generalized” annotation group and colored. **(B)** Principal component analysis comparing the relative changes in protein concentrations in G1 cells of different sizes and asynchronously proliferating cells treated with a saturating dose of rapamycin for 48 h. Exp #1 and Exp #2 are two biological replicates for each experiment. **(C)** Comparison of rapamycin-induced protein concentration changes and size-scaling slopes for the components of the first (PC1, left plot) and second (PC2, right plot) principal components, determined in **(B)**. The size of each dot, corresponding to an individual protein, is proportional to the weight coefficient of this protein within the principal component (see Supplementary Tables S3, S4) for annotated lists of PC1 and PC2 components, respectively).

After observing the differential response of senescence markers to cell size and rapamycin, we next investigated the corresponding proteome response. To do so, we used 2D annotation enrichment analysis (Cox and Mann, 2012) to identify groups of significantly changing proteins and to visualize the degree of their change in both experiments (Figure 6A; Supplementary Table S2) (See Methods for a description of Enrichment Score).

As expected, rapamycin treatment resulted in the significant downregulation of proteins involved in translation, including the ribosome itself. While the translation-associated proteins also become less concentrated in large cells, the degree of change is not as great when compared to the direct inhibition of mTOR (Figure 6A, red dots). Conversely, the decrease in the concentration of DNA- and chromatin-associated proteins was more profound in large cells when compared to rapamycin-treated cells (Figure 6A, blue dots), which is consistent with the concentration of chromatin-associated proteins being more dependent on the concentration of DNA than on the relative growth rate (Claude et al., 2021; Swaffer et al., 2021). Unexpectedly, proteins involved in the actin cytoskeletal regulation were specifically upregulated in rapamycin-treated cells (Figure 6A, light blue dots), whereas proteins that regulate ion homeostasis increased with cell size but were largely unaffected by mTOR inhibition (Figure 6A, light green dots).

As a final way to further explore the similarities and differences in the way rapamycin and increasing cell size remodel the proteome, we performed a combined principal component analysis for the relative protein concentration changes in both experiments (Figure 6B and Supplementary Figure S4B). The first principal component (PC1) explained 49% of the variance and correlated directly with both the increasing cell size and the duration (0, 24, 48 h) of rapamycin exposure (Figures 6B,C (left panel); see Supplementary Table S3 for the detailed list of PC1 components). Interestingly, the two experiments were anti-correlated with one another in the second principal component (PC2), which explained ∼20% of the variance (Figure 6B). The second principal component is therefore enriched for proteins whose concentrations change in opposite directions in larger cells and in rapamycin-treated cells (Figure 6C (right panel); see Supplementary Table S4 for the detailed list of PC2 components). A gene ontology analysis highlighted mitochondrial components, transmembrane ion transporters, translation machinery, and non-coding RNA-related processes as enriched among these differentially modulated proteins.

## Discussion

As cells grow larger, the total protein amount increases in proportion to cell volume so that total protein concentration remains nearly constant (Berenson et al., 2019) (Figure 1A). While it had long been thought that the concentrations of individual gene products also remain constant as cells grow larger (Zhurinsky et al., 2010; Padovan-Merhar et al., 2015), we recently identified size-dependent changes in the concentrations of thousands of individual proteins (Lanz et al., 2022). Because even small changes in the concentration of so many proteins will likely impact cell physiology, this finding led to the new expectation that cell size should affect many aspects of cell physiology.

While the importance of cell size for cell physiology is becoming increasingly appreciated (Demidenko and Blagosklonny, 2008; Miettinen and Björklund, 2016; Neurohr et al., 2019; Cheng et al., 2021; Lengefeld et al., 2021; Wilson et al., 2021; Lanz et al., 2022), the mechanisms through which cell size affects different processes in the cell are largely unknown. Here, we sought to understand more about the origins of size-dependent changes to protein concentrations across the proteome. Since large cells are characterized by a diminished capacity for biosynthesis per unit volume, i.e., growth rate (Cadart et al., 2018; Neurohr et al., 2019; Liu et al., 2022), it was not clear from our earlier study if size-dependent gene expression simply reflected a cell’s declining growth rate or its increasing cell size *per se*. To explore the potentially interdependent effects of cell size and cell growth rate on protein size-scaling, we isolated the effects of decreased cell growth rate by inhibiting mTOR in asynchronously dividing cells. We found that rapamycin-mediated mTOR inhibition, despite modestly reducing the cell size (Supplementary Figure S2), remodeled the proteome in a manner similar to increasing cell size (Lanz et al., 2022) (Figure 3). The striking similarity of these measurements suggests that changes to the proteome in larger cells are associated with the fact that larger cells are unable to scale protein synthesis proportionally with cell volume. It is also possible that cell size drives other changes to the morphology and biomechanical properties of the cell that can also modulate gene expression (Battich et al., 2015).

Since the proteomic changes caused by increasing cell size and by rapamycin treatment were similar, one possibility was that the proteomic effects of cell size were due to its effect on mTOR activity. To test this, we exposed cultured cells to rapamycin for 2 days to inhibit mTOR signaling, and then measured their protein synthesis rates and the proteomes. Although the overall protein synthesis rate decreased about two-fold upon rapamycin treatment, the larger rapamycin-treated cells still had a lower protein synthesis rate per unit volume than their smaller counterparts, and the size-scaling of the proteome was largely unaffected by rapamycin treatment (Figures 4C,D). This result indicates that while we can experimentally decrease the cellular growth rates by treating the cells with rapamycin, it seems unlikely that the naturally occurring size-dependent decrease in cell growth rate and the resulting proteome rearrangements are mediated by the mTORC1 signaling. Instead, they are likely to be mediated by the changes in the DNA-to-cell volume ratio, with DNA becoming a limiting factor in large cells (Figure 4E). However, it remains unclear how changes in the DNA-to-cell volume ratio drive changes to the proteome.

That increasing cell size and rapamycin treatment drive similar changes to the proteome is surprising since large cell size promotes cellular senescence while rapamycin protects against senescence (Demidenko and Blagosklonny, 2008; Demidenko et al., 2009; Leontieva and Blagosklonny, 2014; Lengefeld et al., 2021; Wilson et al., 2021; Crozier et al., 2022; Lanz et al., 2022). Rapamycin treatment results in the upregulation of certain proteins commonly used as senescence markers, including β-galactosidase. This raises the question as to how rapamycin inhibits cell senescence when it drives senescence-like changes to the proteome. One possibility is that the effects of rapamycin in damaged or cell cycle-arrested cells are different from those observed when normally proliferating cells were treated with this drug. In the case of damaged cells, slowing down pathological cell enlargement may have a protective effect that outweighs the senescence-like gene expression changes caused by the slowdown in biosynthesis. A second possibility is that it is the minority of proteins that are expressed differently in large cells and rapamycin treated cells that are the key drivers of cellular senescence (Figure 6 and Supplementary Figure S4B, Supplementary Table S3). Certainly, further investigation of the relationship between the onset of senescence, cell size and growth rate is required for understanding the mechanistic origins of both cellular senescence and differential size scaling across the proteome.

## Data Availability

The datasets presented in this study can be found in online repositories. The mass spectrometry proteomics data have been deposited to the ProteomeXchange Consortium *via* the PRIDE partner repository with the dataset identifier PXD035769.

## Supplementary material

Supplementary Figure S1 The effect of different doses of rapamycin on the proteome after 48 h of treatment. Comparison of PC1 vs. the dose of rapamycin indicates that the mTOR inhibition is saturated at 1 nM. Dashed line corresponds to where the 1 nM dose lies in the first principal component.

Supplementary Figure S2 The effect of rapamycin on cell size. **(A)** Cumulative cell volume distributions of asynchronously proliferating RPE-1 cells exposed to 20 nM rapamycin for 0, 24, or 48 h. **(B)** Cumulative cell volume distributions of asynchronously proliferating RPE-1 cells exposed to 0, 1, 5, 20, or 100 nM rapamycin for 48 h. Cell volume distributions were measured using a Coulter counter. The distribution plots show that rapamycin treatment for 48 h at a saturating dose has a minimal effect on cell size.

Supplementary Figure S3 Cell cycle phase distributions of RPE-1 cells treated with rapamycin. RPE-1 cells were treated with DMSO (48 h) or rapamycin (24, 48 h), then stained with DNA dye Hoechst 33342 and measured by flow cytometry. 50,000 cells were analyzed and plotted for each condition.

Supplementary Figure S4 Comparison of size-dependent and growth-rate-dependent changes to the cell proteome. **(A)** Left plot: Increasing measurement confidence (peptide measurements per protein for both experiments) increases the correlation between the measured size-dependent and rapamycin-dependent changes in protein concentrations (Figure 3B). Right plot: Increasing requirement for measurement confidence decreases the number of proteins considered in the correlation. **(B)** Principal component analysis comparing the relative changes in protein concentrations in G1 cells of different sizes and asynchronously proliferating cells treated with a saturating dose of rapamycin for 48 h. Tables S3 and S4, containing annotated lists of PC1 and PC2 components, respectively, were generated from the principal component analysis with a measurement requirement of at least 8 peptides per protein.
