# Supplementary material for: Delineation of proteome changes driven by cell size and growth rate
Source: Front Cell Dev Biol. 2022 Sep 5;10:980721. doi: 10.3389/fcell.2022.980721 (PMC9483106; doi:10.3389/fcell.2022.980721)
Supplement: Supplementary file 5 [file Image1.pdf]

Supplementary Figures

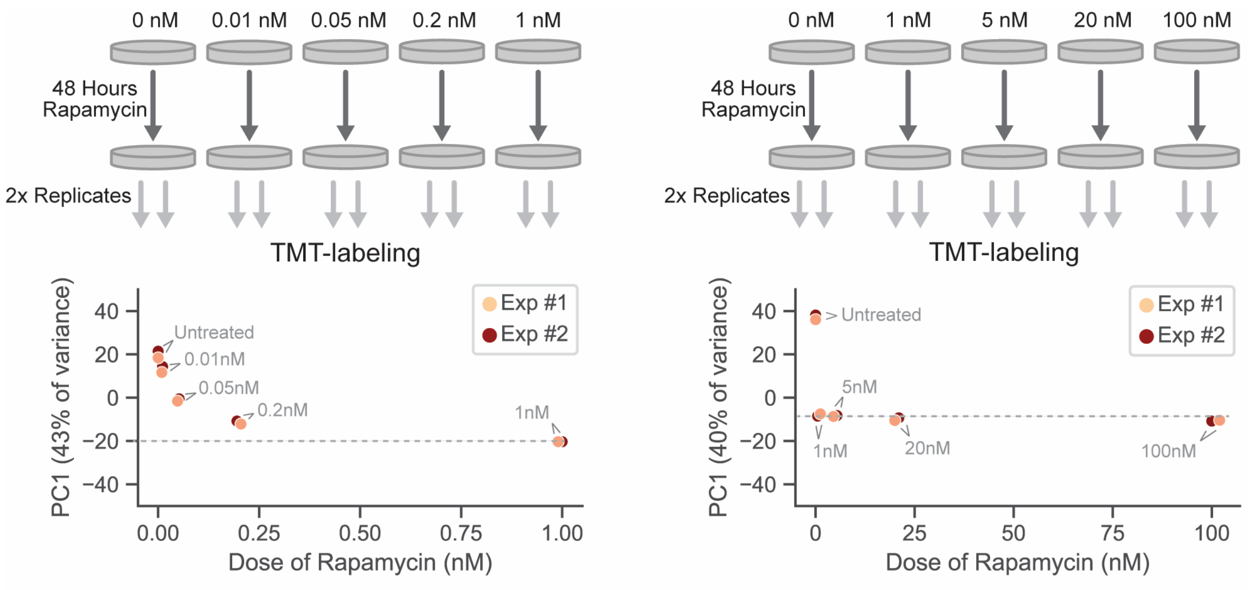

Figure S1: The effect of different doses of rapamycin on the proteome after 48 hours of treatment. Comparison of PC1 vs the dose of rapamycin indicates that the mTOR inhibition is saturated at 1nM. Dashed line corresponds to where the 1nM dose lies in the first principal component.

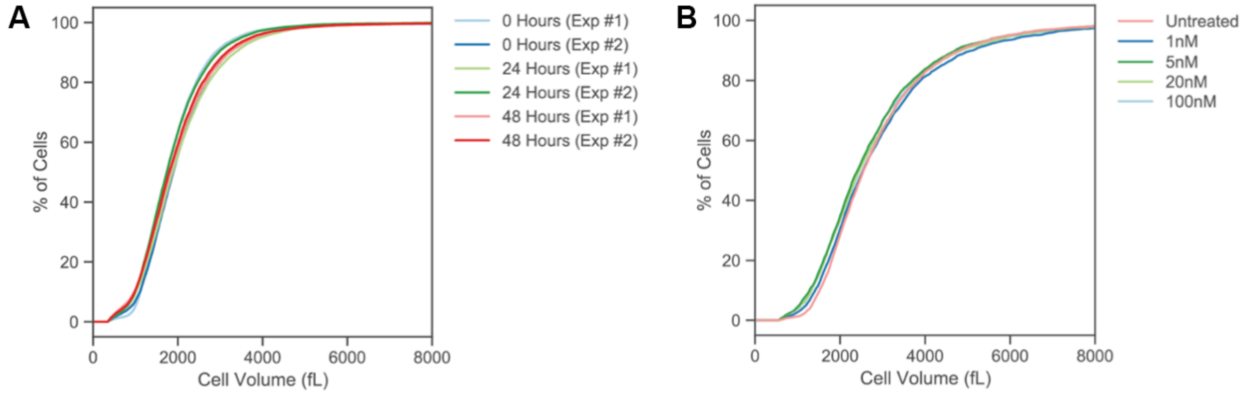

Figure S2: The effect of rapamycin on cell size. **(A)** Cumulative cell volume distributions of asynchronously proliferating RPE-1 cells exposed to 20nM rapamycin for 0, 24, or 48 hours. **(B)** Cumulative cell volume distributions of asynchronously proliferating RPE-1 cells exposed to 0, 1, 5, 20, or 100nM rapamycin for 48 hours. Cell volume distributions were measured using a Coulter counter. The distribution plots show that rapamycin treatment for 48 hours at a saturating dose has a minimal effect on cell size.

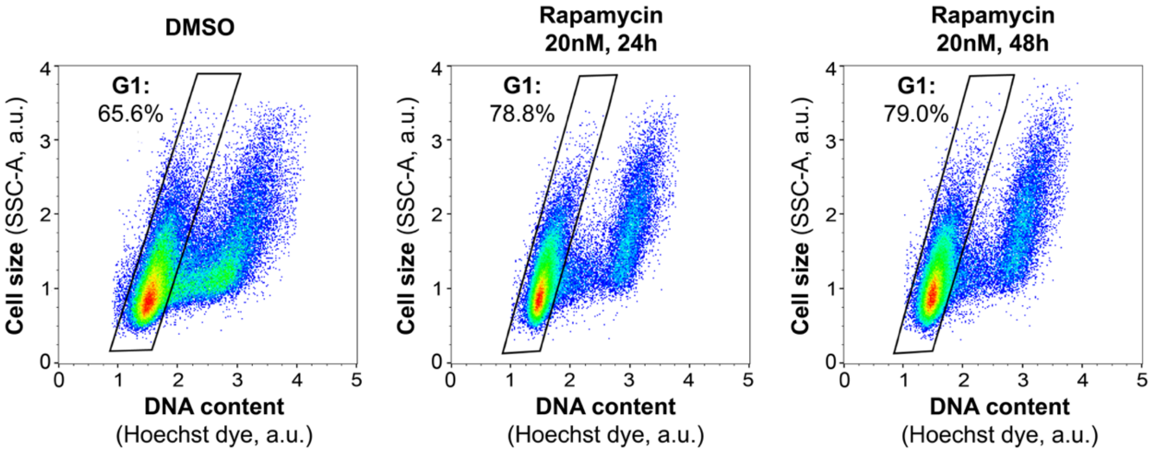

Figure S3: Cell cycle phase distributions of RPE-1 cells treated with rapamycin. RPE-1 cells were treated with DMSO (48h) or rapamycin (24, 48h), then stained with DNA dye Hoechst 33342 and measured by flow cytometry. 50,000 cells were analyzed and plotted for each condition.

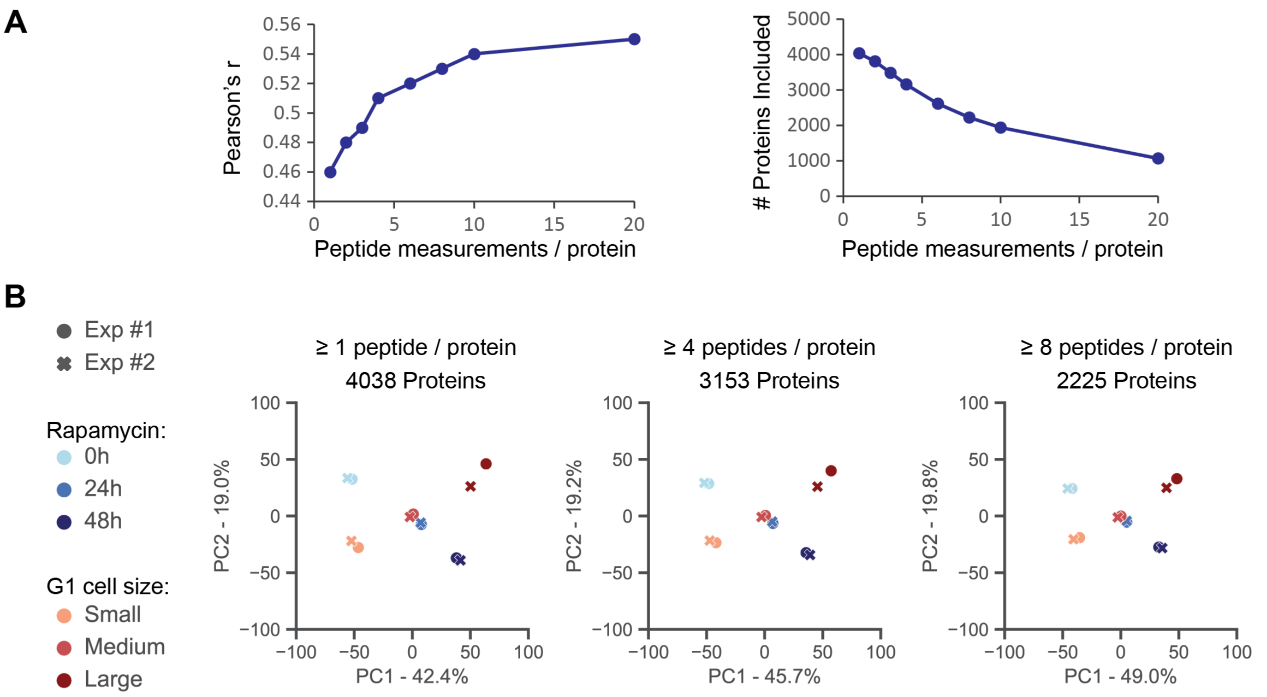

Figure S4: Comparison of size-dependent and growth-rate-dependent changes to the cell proteome. **(A)** Left plot: Increasing measurement confidence (peptide measurements per protein for both experiments) increases the correlation between the measured size-dependent and rapamycin-dependent changes in protein concentrations (**Figure 3B**). Right plot: Increasing requirement for measurement confidence decreases the number of proteins considered in the correlation. **(B)** Principal component analysis comparing the relative changes in protein concentrations in G1 cells of different sizes and asynchronously proliferating cells treated with a saturating dose of rapamycin for 48 hours. **Tables S3** and **S4,** containing annotated lists of PC1 and PC2 components, respectively, were generated from the principal component analysis with a measurement requirement of at least 8 peptides per protein.
